# Died

**Published:** 1866-04

**Authors:** 


### DIED.

On the 14th of March, Dr. D. A. Wilder, in Jackson Mich., of inflammation of the brain. Dr. W. was a member of the graduating class in the Ohio Dental College at the last session. He was a man of rare ability and most estimable character. All who knew him entertained for him the highest regard ; indeed none knew him but to love him. In the prosecution of his studies there was none to excell him. And more than all this he was a good man. In him the profession has lost a talented and interested member, one who promised to be a bright ornament.

We sympathize deeply with her who has sustained the greatest loss, and weeps for the departed loved one.

